# Plain x-ray films in soft tissue infections

**DOI:** 10.11604/pamj.2017.26.149.11393

**Published:** 2017-03-15

**Authors:** Diogo Carrola Gomes, Luísa Quaresma

**Affiliations:** 1 Serviço de Cirurgia 1, Centro Hospitalar Lisboa Central, Rua José António Serrano, 1150-199 Lisboa, Portugal

**Keywords:** Gas gangrene, x-ray, clostridium, soft tissue

## Image in medicine

A 39-year-old male with a history of cocaine abuse presents to the emergency room with pain in the right leg and fever (39ºC) 6 days after injecting himself in the thigh with midazolam pills diluted in tap water to control symptoms of withdrawal. Physical examination revealed rubor, swelling and tenderness of the thigh without crepitation, suggestive of cellulitis. Laboratory findings consisted of a WBC of 10.700x10^6^/L (86% Neutrophiles) and CRP of 170 mg/dL. An X-ray of the leg was performed, revealing a feathering pattern of gas within the soft tissue of the thigh indicative of gas gangrene. Intraoperatively, we found wide necrosis of the muscles and fatty tissue of the posterior thigh without substantial quantities of pus. An extensive debridement was performed and penicillin and clindamycin were administered for two weeks. Cultural exams revealed the presence of aerobic (Streptococcus mitis and Streptococcus oralis) and anaerobic bacteria (Clostridium clostridioforme and Prevotella oralis). The infection was controlled after further daily debridement and the wound was closed with the assistance of a negative-pressure wound therapy device. The patient was discharged from the hospital to a physical rehabilitation facility and regained most of his functional capacity. Gas gangrene is a life-threatening condition, misdiagnosis and failure of recognition in its early stages can prove fatal. Plain X-ray films are readily available in most hospitals and can provide significant information in patients with soft tissue infections where 'time is tissue'.

**Figure 1:**
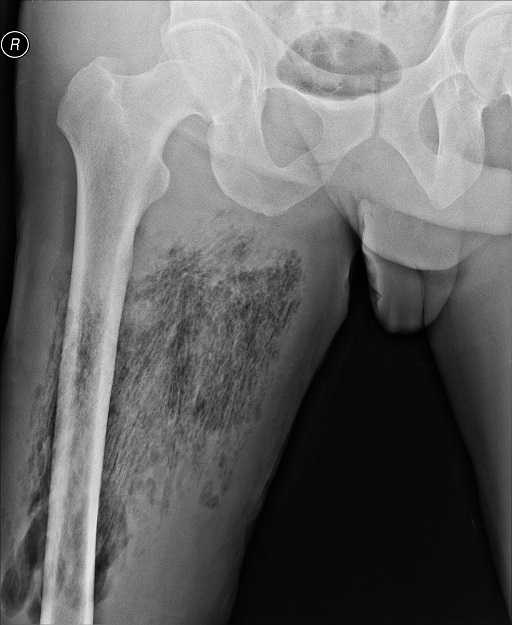
Plain x-ray of the thigh demonstrating a feathering pattern indicative of the presence of gas in the soft tissue

